# Integrative Transcriptomics Data Mining to Explore the Functions of *TDP1α* and *TDP1β* Genes in the *Arabidopsis thaliana* Model Plant

**DOI:** 10.3390/genes14040884

**Published:** 2023-04-09

**Authors:** Paola Pagano, Andrea Pagano, Stefano Paternolli, Alma Balestrazzi, Anca Macovei

**Affiliations:** Department of Biology and Biotechnology “L. Spallanzani”, University of Pavia, 27100 Pavia, Italy

**Keywords:** *Arabidopsis thaliana*, DNA damage, DNA repair, gene expression, tyrosyl-DNA phosphodiesterase 1, stress response

## Abstract

The tyrosyl-DNA phosphodiesterase 1 (TDP1) enzyme hydrolyzes the phosphodiester bond between a tyrosine residue and the 3′-phosphate of DNA in the DNA–topoisomerase I (TopI) complex, being involved in different DNA repair pathways. A small *TDP1* gene subfamily is present in plants, where *TDP1α* has been linked to genome stability maintenance, while *TDP1β* has unknown functions. This work aimed to comparatively investigate the function of the *TDP1* genes by taking advantage of the rich transcriptomics databases available for the *Arabidopsis thaliana* model plant. A data mining approach was carried out to collect information regarding gene expression in different tissues, genetic backgrounds, and stress conditions, using platforms where RNA-seq and microarray data are deposited. The gathered data allowed us to distinguish between common and divergent functions of the two genes. Namely, *TDP1β* seems to be involved in root development and associated with gibberellin and brassinosteroid phytohormones, whereas *TDP1α* is more responsive to light and abscisic acid. During stress conditions, both genes are highly responsive to biotic and abiotic treatments in a time- and stress-dependent manner. Data validation using gamma-ray treatments applied to *Arabidopsis* seedlings indicated the accumulation of DNA damage and extensive cell death associated with the observed changes in the *TDP1* genes expression profiles.

## 1. Introduction

One of the challenges that living organisms face is to respond promptly to genotoxic stress and avoid the accumulation of DNA damage. Maintenance of genome integrity is required for the proper development and faithful transmission of genetic information to the next generations. However, cells are continuously subjected to DNA damage, which is caused by either endogenous factors or exogenous stimuli. Hence, plants have evolved highly efficient mechanisms to detect and repair DNA damage to maintain genome stability [1,2,3,4].

Among the different types of DNA damage, strand breaks (single-SSB and double-DSB) are the most damaging and can be transiently induced by the cell under certain conditions [5,6]. Specific enzymes called topoisomerases I (TopI) produce SSBs to regulate the DNA topology. To accomplish their function, these enzymes form DNA-enzyme covalent intermediates, which are quickly broken at the end of the reaction. Modifications in DNA and the presence of specific drugs such as camptothecin (CPT) can cause the formation of stabilized covalent complexes of enzyme–DNA, posing a risk to genome integrity [7,8]. Tyrosyl-DNA phosphodiesterase 1 (TDP1) acts to disrupt these covalent complexes, releasing TopI. TDP1 was first identified in yeast [9,10] and mammals [11]. The human TDP1 (hTDP1) is a nuclear protein composed of two domains: an N-terminal regulatory domain necessary for recruitment at damaged sites, and a C-terminal catalytic domain [7]. The hTDP1 protein is characterized by two HKD (histidine, lysine, aspartate) motifs containing the conserved amino acid residues of the active site [7,12]. During the TDP1 reaction, a histidine residue (His-263) is responsible for the nucleophilic attack against the phosphorus atom linking the catalytic tyrosine of TopI and the 3′-oxygen of the nucleotide. The TDP1 enzyme binds to DNA, replacing the tyrosine residue. As a result, a TDP1–DNA covalent complex is formed. Subsequently, another histidine (His-493) activates a water molecule, with the consequent hydrolysis of the His-263–DNA bond and the release of the enzyme [11,13].

In plants, the TDP1 function was characterized in *Arabidopsis thaliana* by Lee et al. [14]. Loss-of-function *AtTDP1* mutation resulted in a dwarf phenotype with defects in vegetative and flowering development and reduced fertility. The *A. thaliana tdp1* mutant was hypersensitive to CPT [14], as previously observed in *Saccharomyces cerevisiae* [15]. Subsequent studies of the AtTDP1 enzyme showed that the HKD motifs are critical for its activity, and vanadate (inhibitor of phosphoryl transfer reactions) treatments repress its function [16], suggesting a conserved function in the human and yeast homologues. However, a peculiarity of the plant system is that a small *TDP1* gene subfamily (composed of two members, *TDP1α* and *TDP1β*) has been identified [17] and a recent phylogenetic study reported that *TDP1β* is specific to the plant kingdom [18]. In terms of the amino acid sequence, the two TDP1 proteins contain the conserved fork-head associated domain (FHA) and HKD catalytic sites, while TDP1β contains an additional HIRAN (HIP116, Rad5p, N-terminal) domain, localized between the two HKD catalytic motifs [17,19]. The FHA-HIRAN-HKD sequence organization is present in other eukaryotic nuclear proteins with known functions in mediating phosphorylation-dependent protein–protein interactions [20,21]. The structure of the HIRAN domain was reported in several proteins [22,23], and its function has been related to the recruitment of DNA repair and chromatin remodeling enzymes to specific DNA sites, mainly at stalled replication forks and post-replication damage, possibly acting as a sensor of DNA damage checkpoints [21].

The human TDP1 plays a role in different DNA repair pathways [24]. Likewise, in plants, TDP1α (the canonical homologue of hTDP1) is mainly related to DNA–protein crosslink (DPC) repair [25] and base excision repair (BER) [26] pathways. Using specific single and double mutants of *A. thaliana*, Enderle et al. [25] demonstrated that TDP1(α) acts as a backup for the DNA endonuclease MUS81 and the protease WSS1A in DPC repair. Other studies using *Medicago truncatula TDP1α*-depleted plants revealed different levels of transcriptional modulation (up- and down-regulation, alternative splicing, activation of alternative promoter) of genes involved in DNA damage sensing, DNA repair (mainly BER), and chromatin remodeling, indicating an important role in maintaining genome integrity [26,27].

As for the *TDP1β* gene, this is far less characterized compared to *TDP1α*. In *M. truncatula* (barrel medic), it was shown to be ubiquitously expressed in all plant tissues and developmental stages [17,28]. Moreover, the *TDP1β* gene was differentially expressed upon exposure to NSC120686 (inhibitor of hTdp1 acting as substrate mimetic [29]), being downregulated in calli and upregulated in young seedlings [30,31], possibly suggesting its implication in different developmental aspects. In both *A. thaliana* and barrel medic plants subjected to multiple abiotic stress conditions (cold, heat, salinity, osmotic stress, and UV-B), *TDP1β* was highly upregulated at the earliest timepoints (0.5–1 h) following exposure to stress [19]. More recently, a comparative data mining study using different species (*Selaginella moellendori, Zea mays, Oryza sativa, M. truncatula, Glycine max, Solanum lycopersicum, S. tuberosum*) presented evidence of tissue- and stress-specific responses [18].

Despite the recent literature, the precise roles of the *TDP1* genes in plant development and stress responses are still unclear, especially concerning the non-canonical *TDP1β* gene. To address this gap in knowledge, the current study was designed for an in-depth inquiry into the function of the *TDP1* genes by taking advantage of the abundance of transcriptomics data and bioinformatic platforms available for the model plant *Arabidopsis thaliana*. Hence, an integrative data mining approach was carried out to collect information regarding the *A. thaliana TDP1α* and *TDP1β* gene expression using platforms where RNA-seq and microarray data are deposited. Additionally, to further validate these data, an experimental system consisting of gamma-irradiated *Arabidopsis thaliana* plantlets was implemented and the expression of the two *TDP1* genes comparatively monitored.

## 2. Materials and Methods

### 2.1. Bioinformatics Analyses

Phytozome version 13 (https://phytozome-next.jgi.doe.gov/) was used to retrieve the *Arabidopsis thaliana* TDP1α (Accession No. AT5G15170) and TDP1β (Accession No. AT5G07400) nucleotide and aminoacidic sequences. Gene organization and chromosome localization were obtained from the Phytozome gene browser (https://phytozome-next.jgi.doe.gov/jbrowse/) and ePlant chromosome viewer (http://bar.utoronto.ca/eplant/), respectively. Gene co-expression and co-localization data were retrieved from GeneMania (https://genemania.org/).

The alignment between the two protein sequences was carried out using the ClustalW tool available at https://www.genome.jp/tools-bin/clustalw. Protein–protein putative interaction analysis was conducted using the STRING (https://string-db.org/) online tool. Data from computationally predicted and experimentally documented subcellular localization of the proteins were obtained from ePlant [32,33], as recommended by Winter et al. [34], to generate a confidence score for each distinct subcellular compartment or region. The higher the confidence score for a given subcellular compartment, the more intense the red color in the Cell eFP Browser output.

### 2.2. Gene Expression Data Mining

Data mining was performed using the Bio-Analytic Resource for Plant Biology (BAR) Toronto eFP browser (http://bar.utoronto.ca/efp/cgi-bin/efpWeb.cgi), a visual analytic tool for exploring multiple levels of *Arabidopsis thaliana* data through a user-friendly interface. ePlant (http://bar.utoronto.ca/eplant) is connected to several publicly available web services to download genome, proteome, interactome, transcriptome, and 3D molecular structure data for one or more genes or gene products of interest [35]. Each piece of RNA-Seq or microarray data deposited in this database contains specific references to the study in which data were generated, the types of plant materials, treatments, and data analyses that were used. Mainly, the deposited affymetrix ATH1 array data come from developmental maps [36], seeds [37], developmental mutants [38,39,40,41,42,43], natural variants [44], hormones and chemical treatments [45], the stress atlas [46], and additional stress treatments related to drought [47], selenium [48], gamma-rays [49], and light series [50]. The genes taken into consideration in this data mining approach are *TDP1α* (Accession No. AT5G15170) and *TDP1β* (Accession No. AT5G07400). The selected mode for all the species was “absolute”, indicating that the expression levels for each type of sample/condition were directly associated with the most intense signal recorded for each gene. The mean values were retrieved and used to calculate the fold change (FC) relative to the control for each condition under study.

The obtained data are presented as heatmap models generated using the Shinyheatmap (http://shinyheatmap.com/) application, a software program that generates highly customizable static and interactive biological heatmaps in a web browser [51]. Within this application, the expression values are represented as Z-scores, a numerical measure that describes the relationship of a value with the mean of a group of values. The following parameters were set for all the generated heatmaps: low value—blue color, mid value—white color, high value—red color, apply clustering—none, distance metric—Euclidean, linkage algorithm—complete, apply scaling—row (per gene, not conditions), make trace—none. The Euclidean measure (differences in gene expression values) is used in many conventional algorithms.

### 2.3. Plant Material and Growth Conditions

Seeds of *A. thaliana* wild-type Col0 and *sog1* mutant lines were provided by Dr. Cécile Raynaud (Institute of Plant Sciences Paris-Saclay, IPS2, Orsay, France). For each line, seeds were sterilized with 70% EtOH and 50% bleach as follows: seeds were suspended in a tube containing 50% bleach and, after bleach removal, seeds were washed with 70% EtOH twice. Subsequently, seeds were washed with sterile H_2_O and sown in Petri dishes containing half MS (Murashige & Skoog) media (Duchefa Biochemie) with 0.6% plant agar (Duchefa Biochemie), stratified at 4 °C for 3 days in the dark, and transferred into a growth chamber at 25 °C in light conditions with a photon flux density of 150 μmol m^−2^ s^−1^, photoperiod of 16/8 h, and 70–80% relative humidity. Ten-day-old seedlings were irradiated with gamma(γ)-rays (total dose 100 Gy), by using a panoramic dry ^60^Co source at room temperature. The Petri dishes were subsequently placed back into the growth chamber and samples were collected at selected intervals (30 min, 3 h, 6 h, 12 h) after the γ-ray treatment for the following molecular analysis.

### 2.4. Single-Cell Gel Electrophoresis (SCGE)

The SCGE, also known as the comet assay, was performed under alkaline conditions to quantitatively measure the levels of DNA damage in 10-day-old *A. thaliana* seedlings, as described by Ventura et al. [52]. For slide preparation, aliquots (600 μL) of 1% low-melting-point (LMP) agarose (Sigma-Aldrich, Milan, Italy) in phosphate-buffered saline (PBS) buffer (140 mM NaCl, 2.7 mM KCl, 10 mM Na_2_HPO_4_, 1.8 mM KH_2_PO_4_, pH 7.4) were equally distributed on 2/3 of each slide, carefully levelled horizontally on the bench. Agarose-precoated slides were then dried overnight at room temperature. To isolate the nuclei, approximately 80 seedlings were transferred in an Eppendorf tube containing 500 μL of TE buffer (0.4 M Tris HCl pH 7.5, 1 mM EDTA pH 8.0) and chilled in liquid nitrogen. The material was placed on an inclined Petri dish on ice and carefully sliced with a sharp razor blade. After the removal of cellular residues by filtration, 300 μL of the nuclear suspension was mixed with 200 μL 1% LMP agarose previously melted and kept at 38 °C in a water bath. Aliquots of 100 μL were then distributed on the precoated slides, covered immediately with a cover slip, and incubated on ice for 5 min under dark conditions. Nuclei were denatured in an alkaline buffer (1 mM Na_2_EDTA, 300 mM NaOH, pH 13.0) for 30 min at 4 °C and then electrophoresed in the same buffer for 20 min at 0.72 V cm^−1^ in a dark cold chamber. Slides were then washed in 0.4 M Tris–HCl pH 7.5 two times for 5 min, rinsed in 70% ethanol (*v*/*v*) three times for 10 min at 4 °C, and dried overnight at room temperature. To evaluate the nuclei’s morphologies, the prepared slides were stained with 4′,6-diamidino-2-phenylindole (DAPI, 20 μL; Sigma-Aldrich, Milan, Italy) and observed with an Olympus BX51 fluorescence microscope with a 100W mercury lamp (excitation filter of 340-380 nm and barrier filter of 400 nm). Nucleoids were classified into 5 classes according to the lengths of their tails, which reflected the extent of the damage, and results were expressed in arbitrary units (a.u.), calculated using the following formula: *a*.*u*. = Σ(N_*c*_ ∙ *c*) ∙ 100/N_*tot*_, where, “N*_c_*” indicates the number of nuclei identified for each class, “N*_tot_*” the total nuclei identified, and “*c*” is the class identification number (0, 1, 2, 3, 4) [53]. For each treatment, three replicated samples were analyzed in two independent experiments.

### 2.5. Diffusion Assay

The DNA diffusion assay was performed as described by Macovei et al. [54] to assess cell viability based on nuclear morphology. The assay is based on the fact that low-molecular-weight DNA fragments generated during programmed cell death (PCD) and necrosis diffuse in the agarose, resulting in an average nuclear size three times higher compared to nuclei from viable cells. PCD nuclei are characterized by a DNA halo without a clear boundary, and this typical morphology results from the diffusion of nucleosomal-sized DNA fragments generated by endonucleolytic cleavage. By contrast, necrotic cell nuclei undergo random fragmentation that produces a clear DNA halo, with a well-defined outer boundary and nonhomogeneous content [54].

The nuclei’s isolation was performed as previously described for the comet assay. After the removal of the cover slip, the slides were immersed in saline lysis buffer (2.5 M NaCl, 100 mM EDTA, 10 mM Tris–HCl pH 7.5) for 20 min in a dark cold (4 °C) chamber. Subsequently, slides were incubated in neutral Tris–Borate–EDTA (TBE, 89 mM Tris Base, 89 mM Boric Acid, 2 mM EDTA, pH 8.3) solution for 5 min three consecutive times in the same cold chamber. The slides were rinsed in 70% ethanol for 5 min and subsequently in absolute (99.8%) ethanol for another 5 min, and finally air-dried overnight. To evaluate the nuclei’s morphology, the prepared slides were stained with 20 μL DAPI and observed with the same Olympus BX51 fluorescence microscope. One hundred nuclei/replicate stained with the fluorescent dye were analyzed for their morphology modifications and included in classes (0, 1, 2) resulting from the passive diffusion of DNA molecules. The level of cell death was scored in a.u. following the same formula as indicated for the SCGE, whereas the percentage (%) of nuclear morphology was also used to represent the different types of cell death reflected.

### 2.6. Quantitative Real-Time Polymerase Chain Reaction (qRT-PCR)

RNA isolation was carried out using the TRIzol^TM^ Reagent (Thermo Fisher Scientific, Monza, Italy) according to the supplier’s indications. A DNase (Thermo Fisher Scientific) treatment was also performed, as indicated by the manufacturer. RNA was quantified using NanoDrop (Biowave DNA, WPA, Thermo Fisher Scientific). Subsequently, cDNAs were obtained using the RevertAid First Strand cDNA Synthesis Kit (Thermo Fisher Scientific), according to the manufacturer’s suggestions. The qRT-PCR was performed with the Maxima SYBR Green qPCR Master Mix (2×) (Thermo Fisher Scientific), according to the supplier’s indications, using a Rotor-Gene 6000 PCR apparatus (Corbett Robotics Pty Ltd., Brisbane, Australia). Amplification conditions were as follows: denaturation at 95 °C for 10 min, and 45 cycles of 95 °C for 15 s and 60 °C for 30 s and 72 °C for 30 s. Oligonucleotide primers were designed using the Real-Time PCR Primer Design program Primer3Plus (https://primer3plus.com) from GenScript and further validated through the online software Oligo Analyzer (https://eu.idtdna.com/calc/analyzer). The following genes were tested: *TDP1α* (Accession No. AT5G15170; FW: 5′-CGGTGACGGAGAGAGAAAGA-3′; RV: 5′-GGACAAAAACGACGAATGGC-3′) and *TDP1β* (Accession No. AT5G07400; FW: 5′-TCACCTTGTTGCTTCAGTGC-3′; RV: 5′-CCAGTCGTTTCAGTTGTGCT-3′). Relative quantification was carried out using ubiquitin 4 (Accession No. AT5G20620; FW: 5′-TCATTTGGTGCTTCGTCT-3′; RV: 5’-GTCTCTGCTGATCTGGTG-3′) as a reference gene [55]. The raw, background-subtracted fluorescence data provided by the Rotor-Gene 6000 Series Software 1.7 (Corbett Robotics) were used to estimate the PCR efficiency (E) and threshold cycle number (Ct) for each transcript’s quantification, while the Pfaffl method [56] was applied to calculate the transcript’s relative quantification. All reactions were performed in triplicate.

### 2.7. Statistical Analyses

For all data, statistically significant differences were determined using Student’s *t*-test. Means were considered statistically different when *p* ≤ 0.05 and are indicated with asterisks.

## 3. Results and Discussion

### 3.1. Arabidopsis TDP1α and TDP1β Have Different Sequence Organizations and Predicted Interactions

A preliminary in silico analysis was carried out comparatively for the *TDP1α* and *TDP1β* genes and protein sequences. The alignment between the two protein sequences indicates that only 123 aa are conserved, mainly located within the FHA domain and the catalytic HKD sites, translated into approximatively 11% similarity between TDP1α (606 aa) and TDP1β (1085 aa) (Figure S1). Both genes are localized on chromosome 5 (Figure S2a,b), and while TDP1α’s genomic sequence is of 3409 nucleotides organized in 14 exons, the TDP1β sequence has 4817 nucleotides divided into 12 exons (Figure S2a). A gene co-expression prediction using the GeneMania application indicates that *TDP1α* is mostly co-expressed with genes involved in abiotic (*AT1G61240, AT1G68820*), biotic (*AT1G48430, AT5G08430, AT1G60860*), and genotoxic (*ZEU1, MTP1, XPD*) stress responses, as well as with genes involved in light (*AT1G06630, PHYC, XPD*) and brassinosteroid (*MNS4*) signaling (Figure S3a). Nonetheless, the putative protein–protein interaction analysis shows that the majority of interacting proteins are involved in different DNA repair pathways, such as BER (AT3G14890, LIG6), NER (APTX), DPC repair (MUS81), DNA distortions (Top1α, Top1β), or DSB (KU70, KU80) repair (Figure S4a). Differently, the *TDP1β* gene co-expression network includes genes mostly involved in DNA binding (*AT3G21430, AT4G15730, HDG9, HDG2, ATHB-14*), DNA repair (*FAN1, AZG2, MCM10*), molecule transport (*AT1G53660, AT4G75920, AT1G75920*), and biotic stress (*AT3G14580, AT5G15300, AT5G42840, AT3G13225, PCMP-E47, LECRK81, ULP1B*) (Figure S3b). On the other hand, the putatively predicted protein–protein interactions include mainly proteins involved in DNA binding (AT5G51300, AT5G46630, HPPBF-1, OBP1) and DNA damage tolerance (RAD5) (Figure S4b). When considering the computationally predicted and experimentally documented subcellular localizations of the two proteins, the ePlant browser indicates that while TDP1α seems to be mostly spread in the cytoplasm (Figure S5a), TDP1β is largely found in the nucleus (Figure S5b). A previous study looking into promoter in silico analyses evidenced that the two gene promoters are rich in stress-related *cis*-elements, with light- and hormone-responsive elements being the most abundant [19]. Considering the elements specific to each promoter region, the *Tdp1α* gene promoter contains many elements involved in abscisic acid (ABA) responses, lignin biosynthesis, and light responses. The detected elements specific to *Tdp1β* include *cis*-elements involved in the gibberellin (GA) response, differentiation of mesophyll cells, and control of plant morphology [19].

The preliminary predictions gathered here can help to better discuss the following data relative to the comparative expression of the two genes in the multiple systems available for *A. thaliana* transcriptomics datasets mined in the current study.

### 3.2. Arabidopsis TDP1α and TDP1β Comparative Expression during Plant Development and Ecotype Genetic Backgrounds

**Figure 1:**
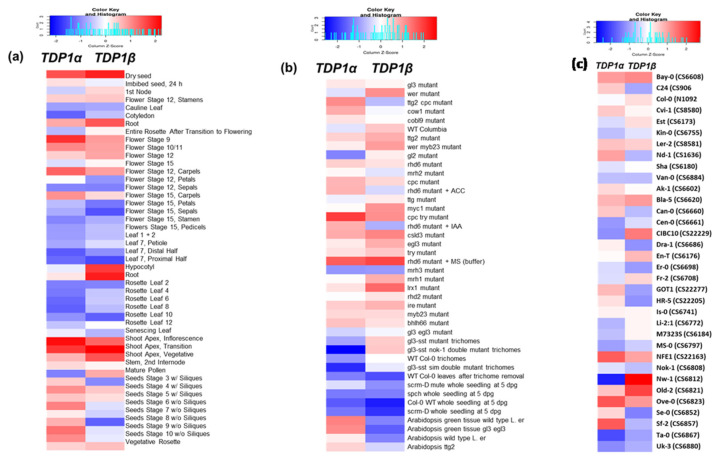
Heatmap representation of *TDP1α* and *TDP1β* gene expression obtained for (**a**) developmental map, (**b**) developmental mutant, and (**c**) natural variant ecotype collections.

To evaluate the expression patterns of the two *TDP1* genes, the first focus was placed on the different types of tissues, development stages, developmental defects (mutants), and ecotypes. The “developmental map” data present the *TDP1α* and *TDP1β* gene expression retrieved from the dataset published by Schmid et al. [36] (Figure 1a). Analyzing the pattern of expression shown in the heatmap, it is possible to observe that mostly the genes are similarly expressed, having generally low expression values in flowers and rosettes and high expression values in shoot apexes. This is in agreement with data from other species showing that both genes are highly expressed in shoot apical meristems [18], probably because these specific stem niches have to rapidly and promptly repair any type of DNA damage to avoid its propagation to generating tissues [57]. Focusing on the divergent expression between the two genes, it is possible to observe that the *TDP1β* gene is more expressed than *TDP1α* in roots and hypocotyls. This indicates that the function of this gene could be more required in these tissues.

To better investigate the involvement of the two genes in plant development, a collection of *A. thaliana* mutants, mainly considering mutations affecting specific phenotypes, was evaluated (Figure 1b). Considering the materials tested in this analysis, guard cell meristemoids [41], trichomes [38,39,40], and root epidermidis were sampled from Col-0 and mutants grown in vitro [42,43]. Looking at the heatmap, it is possible to observe that in the root epidermidis, *TDP1β* is more expressed than *TDP1α* in the *wer* mutant. The Weekly Epidemiological Record (*WER*) gene encodes a nuclear-localized MyB-related protein involved in root and hypocotyl epidermal cell fate determination and its loss of function produces a phenotype with extra root hairs [58]. These results may support the hypothesis of the possible involvement of *TDP1β* in root and hypocotyl development, in agreement with what was observed above (Figure 1a). Other mutants worth mentioning (with differential expression of the *TDP1α* and *TDP1β* genes) are *gl2* and *gl3 egl3* mutants, along with *cpc* and *ttc*, all being involved in the complex pathway of root hair formation. The data mining analysis showed that these mutants are characterized by a different pattern of expression of the two *TDP1* genes. In fact, in the *gl2* mutant, *TDP1α* is downregulated while the *gl3 egl3* double mutant shows upregulation of the gene, a contrasting behavior compared to *TDP1β* (Figure 1b). This may indicate that the two genes may be differentially regulated in relation to the root hair formation pathway. Root hair cell fate is regulated by a transcription factor complex that promotes the expression of the homeodomain protein GLABRA 2 (GL2), which blocks root hair development; this is also influenced by hormonal regulation and mainly brassinosteroids [59]. This complex also includes WER, GL3, Enhancer of GL3 (EGL3), and TRANSPARENT TESTA GLABRA 1 (TTG). GL2 blocks the formation of root hairs by inhibiting Root Hair Defective 6 (RHD6). The suppression of GL2 triggers epidermal cells to enter into the root hair cell fate with the activation of RHD6. Here, both genes are highly expressed in the *rhd6* mutant (Figure 1b). In these mutants, previous works reported that root hair formation is conditioned by nitric oxide (NO) and auxin in terms of cell wall remodeling [60]. Additionally, other reports indicate that the vanadate(V)-induced root hair formation is blocked in the *rdh6* mutants, with a consequent delay in plant growth, suggesting that *RHD6* may be involved in V-induced root hair initiation, probably through ethylene- and auxin-independent pathways [61]. This may be of relevance since, in the *A. thaliana tdp* mutant, vanadate was shown to inhibit the TDP1 enzymatic activity and retarded plant growth [16].

In trichoblasts, the presence of Caprice (CPC), along with GL3, EGL3, and TTG1, suppresses GL2, leading cells to enter the root hair cell fate [59]. The two *TDP1* genes have contrasting behaviors in the *ttg2 cpc* mutant, with *TDP1α* being upregulated and *TDP1β* being downregulated. In addition, considering the *TDP1β* gene expression, it is possible to observe that it is highly expressed in the leucine-rich repeat extensin 1 (*lrx1*) mutant. Previous studies have shown that the inhibition of Target of Rapamycin (TOR) resulted in the suppression of phenotypic defects in the *lrx1* mutant [62]. TOR is involved in the regulation of plant responses to a vast array of signals (e.g., nutrients, hormones, light, stresses, or pathogens) [63]. Due to its involvement in regulating cell division, TOR has been also shown to promote the activity of E2Fa/b transcription factors in activating cell cycle genes, especially in shoot and root apexes [64]. Based on these results, we could speculate about a possible relation between *TDP1β* and TOR concerning hair root formation, but these results will need to be further investigated.

Over 750 natural accessions, or ecotypes, of *A. thaliana* have been collected and deposited in specific databases, such as TAIR. The ecotypes vary in traits such as leaf shape, flowering time, disease resistance, seed dormancy, etc., and are generally used by the research community to uncover the genetic interactions that underlie plant responses to environmental conditions or the evolution of different morphological traits. Hence, the gene expression patterns of *TDP1α* and *TDP1β* were also examined in a collection of ecotypes to evaluate whether their profiles may change in these different genetic backgrounds (Figure 1c). The data analyzed were retrieved from aerial parts of 4-day-old seedlings grown in a greenhouse at 23 °C under continuous light [44]. The collected data indicate high variability in the expression profiles of the two genes among the different ecotypes. The upregulation of *TDP1α* is observed in the ecotypes NFE1 (CS22163, from UK), Ove-0 (CS6823, from Ovelgoenne, DE), and Sf-2 (CS6857, from San Feliu, ES). NFE1 is characterized by large rosettes with numerous leaves and vernalization at the rosette stage to induce flowering, while Ove-0 has an early flowering time, presenting epinastic leaves (downward bending) as a result of disturbances in their growth, and Sf-2 exhibits a late flowering behavior, but it can also be induced to flower early by low-temperature treatment [65]. An evident contrasting behavior among the two *TDP1* genes is seen in the Nw-1 (CS6812, from Neuweilnau, DE) ecotype, where *TDP1β* is upregulated and *TDP1α* is downregulated. This ecotype is phenotypically characterized by leaf margins slightly serrated and it requires at least one week of cold treatment for optimal germination; hence, it requires longer treatments to break seed dormancy.

Summarizing these results, the *TDP1β* gene could have a role to play in root development; these data are supported also by the observations related to defective mutant collections, indicating its possible involvement in pathways related to root hair formation. As for the data coming from different *Arabidopsis* ecotypes, at this point, it is difficult to draw any meaningful conclusion, but future studies could be designed to possibly correlate the presence of specific SNPs with the gene expression data.

### 3.3. Arabidopsis TDP1α and TDP1β Comparative Expression in Response to Hormone Treatments

**Figure 2:**
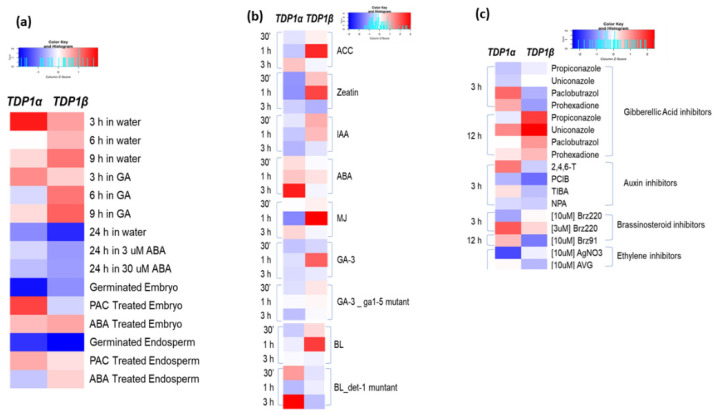
Heatmap representation of *TDP1α* and *TDP1β* gene expression during hormone treatments (**a**) in seeds during the early phases of germination and (**b**) in 7-day-old *A. thaliana* seedlings. (**c**) *TDP1* gene expression patterns in response to hormone inhibitors.

Plants rely on hormones to regulate every aspect of their biology as extensive crosstalk between hormone signaling pathways governs both plant development and stress responses. The expression patterns of *TDP1α* and *TDP1β* were evaluated in response to different hormone treatments (Figure 2) by mining data from studies where these treatments were implemented in *A. thaliana* plants. The plant material investigated came from seeds and 7-day-old plantlets, and the mined data are part of the AtGenExpress project [45]. The antagonistic roles of ABA and GA in the regulation of seed dormancy and germination are well established [66,67]. Additionally, several studies in other plants have already evidenced that the *TDP1* genes change their expression during seed germination, both in the absence/presence of different stress-inducing agents and seed priming treatments [17,28,68,69,70]. Hence, the seed germination process was targeted to evaluate the expression profiles of the *Arabidopsis TDP1α* and *TDP1β* genes, particularly referring to seed imbibition and the involvement of hormones in this process (Figure 2a). Data collected from seeds imbibed in water or with the addition of hormone treatments (5 µM GA and 30 µM ABA) [37] were investigated. The first observation is that *TDP1α* is more expressed than *TDP1β* at 3 h of imbibition, while, at subsequent timepoints (6 h, 9 h), the *TDP1β* gene is more expressed; this is in agreement with experimental data reported in other plants [17,28]. When considering seed imbibition in the presence of different hormone treatments, both *TDP1* genes seem to be upregulated by GA treatments and downregulated by ABA. Since GA is known to promote germination, the upregulation of the genes during this treatment suggests that both genes are required during seed germination, data in agreement with previously mentioned studies [17,28,68,69,70].

When looking at the expression of the two genes in *Arabidopsis* plantlets treated with different hormones (Figure 2b), the *TDP1α* gene is upregulated after 3 h of treatment with ABA, whereas *TDP1β* is upregulated by 1-aminocyclopropane 1-carboxylic acid (ACC), zeatin, methyl jasmonate (MJ), brassinosteroids (BL), and GA. Concerning at least the ABA and GA responses, this is in agreement with the presence of ABA-related *cis*-elements in *TDP1α* and GA elements in the *TDP1β* promoter [19]. When the BL treatment was carried out on *det-1* mutants, the situation was inverted and the *TDP1α* gene was more expressed than *TDP1β*. De-etiolated 1 (DET1) is a key negative regulator of photomorphogenesis that positively regulates the light-induced greening of the etiolated seedlings by repressing ABA responses. DET1 interacts with damaged DNA binding protein 1 (DDB1) to form the COP10-DET1-DDB1 (CDD) complex, which facilitates the degradation of positive regulators of photomorphogenesis, repressing it under dark conditions [71]. The CDD complex negatively regulates ABA responses, leading to the degradation of ABA receptors. It has been hypothesized that ABA coordinates and regulates the greening of seedlings through a process in which three main proteins are involved: DET1, histone deacetylase 6 (HDA6), and far-red elongated hypocotyl 3 (FHY3) [72].

Among GA inhibitors, propiconazole (10 µM), paclobutrazol (10 µM), uniconazole (10 µM), and prohexadione (10 µM) treatments [45] resulted in the upregulation of the *TDP1β* gene at 12 h (Figure 2c). Auxin inhibitor treatments with 2,4,6-trihydroxybenzamide (10 µM), p-chlorophenoxyisobutyric acid (PCIB, 10 µM), and 2,3,5-triiodobenzoic acid (TIBA, 10 µM) resulted in the mild downregulation of *TDP1β*. BL inhibitor treatments with brassinazoles resulted in the upregulation of *TDP1α* at 3 h when Brz220 (3 µM) was used and the downregulation of *TDP1β* at 12 h when Brz91 (10 µM) was used.

Based on the gathered results, it could be hypothesized that the *TDP1α* gene may be mainly related to ABA responses, while the *TDP1β* gene is responsive to multiple hormone treatments, including GA and BLs. The responsiveness of *TDP1β* to BL treatments could also be connected to the putative roles of this gene in root hair formation, as seen in the previous section.

### 3.4. Arabidopsis TDP1α and TDP1β Comparative Expression under Different Light Series

**Figure 3:**
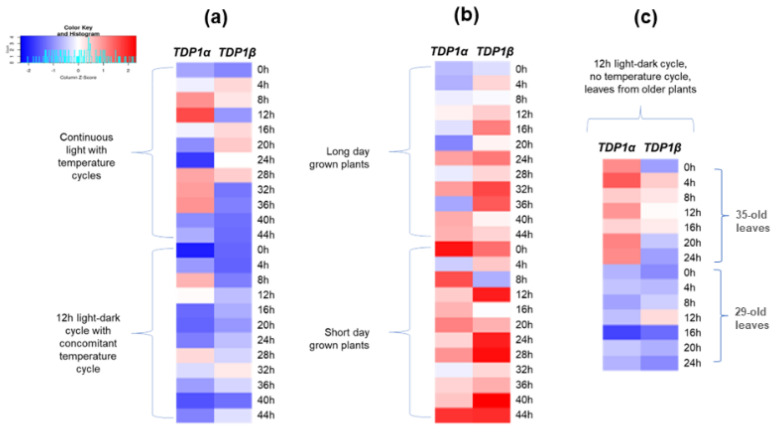
Heatmap representation of *TDP1α* and *TDP1β* gene expression during different light series. (**a**) 7-day-old seedlings under continuous light with temperature cycles and 12 h light–dark cycle with concomitant temperature cycle. (**b**) Seedlings under long-day and short-day conditions. (**c**) Leaves of 35-day- and 29-day-old plants grown in soil under 12 h light–dark cycle, no temperature cycle.

The circadian clock regulates plant physiology and metabolism during the day (24 h). Natural responses to thermal and photo-cycles are essential for plant fitness and some studies suggest that 6% to 36% of the *A. thaliana* transcriptome is under the regulation of the circadian clock [50]. Plants are exposed to daily alternation between light and darkness, along with changes in irradiance, following the regulation of gene expression through red and blue light receptors. Moreover, the metabolic and physiological processes such as photosynthetic carbon fixation in leaves are regulated based on circadian rhythms. The fluctuation of the carbon balance is crucial to the growth of plants; even a few hours of carbon depletion leads to the inhibition of growth, which can be only slowly reversed [73]. To investigate whether the expression of the *TDP1α* and *TDP1β* genes is influenced by the circadian rhythm, the *Arabidopsis* atlas mined data were gathered and represented as a heatmap (Figure 3). Continuous light (100 µE) with temperature cycle treatment (22 °C/12 °C cycles for 12 h each) was monitored, along with a 12 h light–dark cycle with concomitant temperature cycle. Long-day and short-day treatments were performed with 16 h light at an intensity of 90 µE and 8 h at a light intensity of 180 µE, respectively, at 22 °C. Lastly, the 12 h light (180 µE)–dark cycle was carried out in plants of different growth stages (35-day- and 29-day-old) maintained in soil [50].

The hereby performed analysis indicates that *TDP1α* is upregulated by temperature during continuous light conditions but not during a light–dark cycle (Figure 3a). Seedlings grown on long-day and short-day cycles showed the generalized upregulation of both genes, with more intense expression during short-day conditions, maybe caused by the higher energy of the imposed light (Figure 3b). The two experiments with Col-0 leaves treated with a light–dark cycle of 12 h show that *TDP1α* is generally more expressed than *TDP1β* in older leaves (35 days) (Figure 3c). These results may suggest the higher responsiveness of the *TDP1α* gene to light cycles, in accordance with the presence of a high number of light-responsive *cis*-elements in its promoter region [19]. Moreover, recent studies point to a tight ABA–light crosstalk as ABA signaling components interact with multiple light modulators (e.g., photoreceptors, transcription factors, posttranslational modifiers) [74]. Therefore, this could also be the case for the *TDP1α* gene considering its response to both light and ABA treatments.

### 3.5. Arabidopsis TDP1α and TDP1β Comparative Expression during Abiotic and Biotic Stresses

**Figure 4:**
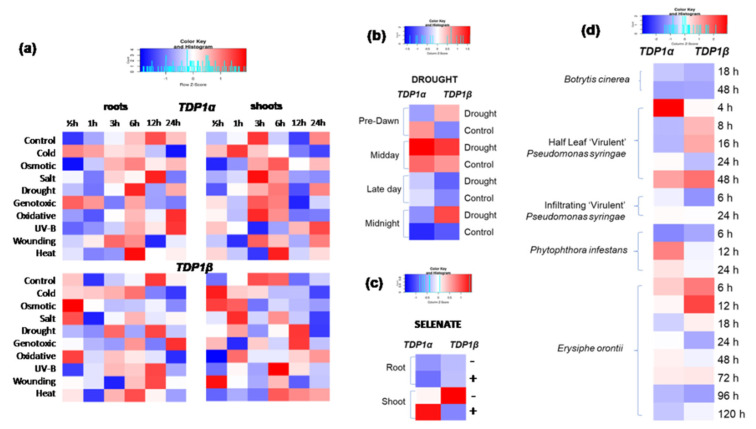
Heatmap representation of *TDP1α* and *TDP1β* gene expression in (**a**) roots and shoots of *A. thaliana* plants subjected to different stress conditions and monitored at several time intervals, (**b**) drought stress, (**c**) selenate treatment, and (**d**) biotic stresses.

The expression of the *TDP1α* and *TDP1β* genes was mined from different abiotic and biotic stress conditions (Figure 4). Data from abiotic stress were gathered from Kilian et al. [46], where *A. thaliana* ecotype Col-0 plants were exposed to the following abiotic stresses: heat (42 °C), cold (4 °C), drought (plants were removed from the growth boxes and exposed to a stream of air in a clean bench for 15 min), salt (NaCl, 250 mM), high osmolarity (PEG6000, 150 g/L), UV-B light (15 min exposure to 280–315 nm), wounding (punctuation of the leaves with a custom-made pin-tool consisting of 16 needles), oxidative (10 μM methyl viologen), and genotoxic (bleomycin 1.5 μg/mL) stress.

As shown in the heatmap (Figure 4a), the initial transcriptional stress reaction involves the upregulation of *TDP1β*, while *TDP1α* tends to be upregulated at later timepoints. It is also possible to observe that *TDP1α* is generally more responsive than *TDP1β,* especially in the aerial parts. Since drought is one of the major environmental factors influencing plant growth and development worldwide, the expression data of the two *TDP1* genes were mined from the *Arabidopsis* atlas, where an additional experimental system was implemented for this type of stress (Figure 4b). The materials used in the study by Wilkins et al. [47] included rosette tissue from the leaves of 32-day-old Col-0 plants. The experimental system contained well-watered and water-stressed (25% water loss) plants sampled at different times during the natural circadian cycle. The data mining results support the previous results (Figure 4a, drought) as both genes were still differentially modulated by drought. Moreover, these data also show that *TDP1β* is mostly upregulated during pre-dawn, midday, and midnight, while *TDP1α* is mostly upregulated only at midday (Figure 4b), thus highlighting the more stringent circadian regulation of the latter. Selenium is an important micronutrient for plant growth since, at low concentrations, it can enhance the plant’s antioxidant defense but, at high concentrations, it becomes damaging, being associated with the overproduction of ROS, leading to growth suppression [75]. *A. thaliana* Wassilewskija ecotype 10-day-old plants were grown in the presence/absence of 40 µM selenate [48]. The gene expression data mining under these conditions indicates that both genes are downregulated in the roots, while the *TDP1α* gene is highly expressed in treated shoots and *TDP1β* is highly expressed in non-treated shoots (Figure 4c). Hence, while *TDP1α* induces by the presence of selenate, *TDP1β* is induced by the lack of this compound. The *Arabidopsis* transcriptome analyses related to selenium stress responses indicate that many genes involved in the ethylene and jasmonic acid signaling pathways were upregulated by these treatments, along with other transcripts generally associated with salt and osmotic stresses [48].

Biotic stress is a major issue, mostly due to pre- and postharvest losses [76]. Within the *Arabidopsis* atlas, biotic stress data gathered from different labs refer to studies using *Botrytis cinerea*, *Pseudomonas syringae*, *Phytophthora infestans,* and *Erysiphe orontii* infected plants. The *B. cinerea* infection was performed on 4-week-old *A. thaliana* (Col-0), whereas the *E. orontii* infection was carried out on leaves of 28-day-old plants. The *P. syringae* treatments were applied in two ways: the half-leaf experiment was carried out by injecting half of a plant leaf while the other was subsequently collected for analysis, while the infiltrating experiment data come from analyzing infiltrated leaves. The *P. infestans* data include plant material from leaves of 5-week-old plants. Data retrieved from all these platforms and represented as a heatmap (Figure 4d) show that the *TDP1α* gene is upregulated by *P. syringae* and *P. infestans*, while the *TDP1β* gene is upregulated by *E. orontii* at early timepoints after treatment, possibly suggesting that each gene may have a role to play during particular types of bacterial infections in a time-specific manner.

Taken together, these data pinpoint once more the involvement of both genes in response to both biotic and abiotic stresses, in accordance with other data [17,18,19,28,68,69], the predicted co-expression data (Figure S3), and the abundance of stress- and hormone-related *cis*-acting elements in their promoter regions [19].

### 3.6. Arabidopsis TDP1α and TDP1β Comparative Expression during Treatments with Cytotoxic and Genotoxic Agents

**Figure 5:**
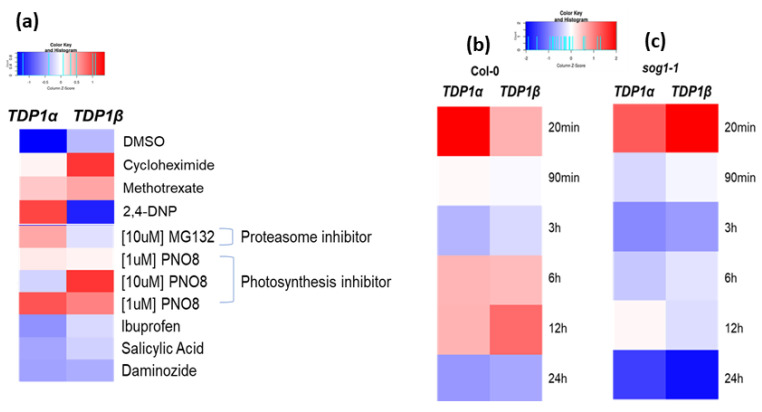
Heatmap representation of *TDP1α* and *TDP1β* gene expression in response to (**a**) different chemical treatments, and (**b**) gamma-rays in Col-0 wild-type and (**c**) *sog1-1* mutants.

Cytotoxic and genotoxic damages can be induced by endogenous and exogenous factors. In this case, different chemical and physical agents were used to induce cytotoxic and genotoxic stresses in seeds and seedlings, and data were mined from different studies where these treatments were implemented in *Arabidopsis* [37,45,49] (Figure 5).

Among the chemical treatments applied to seeds for a period of 24 h (Figure 5a) [37], dimethyl sulfoxide (DMSO, 1%) was used as a control and an agent to dissolve the chemical compounds, even though this compound can impair seed germination and plant development when used in high concentrations [77]. Indeed, the DMSO treatment seems to affect the expression of the *TDP1* genes, in agreement with previous studies from other model systems [31]. Cycloheximide (CHX, 1 µM) strongly induces the expression of *TDP1β*, while 2,4-dinitrophenol (2,4-DNP, 25 μM) treatment acts oppositely on both genes, thus downregulating *TDP1β* and upregulating *TDP1α*. CHX is a naturally occurring fungicide produced by the bacterium *Streptomyces griseus* [78]. It exerts its effects by interfering with the translocation step in protein synthesis, hence blocking the eukaryotic translational elongation [79]. On the other hand, CHX treatments are responsible for the enhanced induction of many stress genes [80], while, in other studies, these were associated with hindered seed germination through the inhibition of protein synthesis [81]. The 2,4-DNP agent is an ATP synthesis uncoupler that inhibits ATP synthesis, and it was shown that its use in plants affected root permeability [82] in relation to auxin and ethylene production [83]. The observed results may indicate that, in seeds, *TDP1α* could be required in situations where stress conditions are related to low ATP, which affects membrane permeability during germination, whereas *TDP1β* could be necessary during transcriptional elongation.

Among the chemical treatments applied to plantlets [45], MG132 was used as a proteasome inhibitor [84] that can alter the production of reactive oxygen species (ROS), as proteasomes are among the direct regulatory mechanisms of ROS production in plants [85]. The gathered results indicate that *TDP1α* is more abundantly expressed compared to *TDP1β*, in agreement with the proposed role of this gene in the BER pathway [26]. Moreover, a photosynthesis PSII system inhibitor treatment was implemented by using the N-octyl-3-nitro-2,4,6- trihydroxybenzamide (PNO8, 1 and 10µM) compound, known to specifically hinder electron transport [86]; hence, it can be also used as an oxidative stress inducer [87]. This treatment resulted in both *TDP1* genes’ upregulation, even at the lowest concentration, pointing again toward relevant roles in ROS-induced stresses [17,28,68,69,70]. The last compounds, ibuprofen (10µM), salicylic acid (10 µM), and daminozide, show mild downregulation in both genes. These treatment effects can be viewed in relation to the fact that pharmaceuticals pose a risk to the environment and their potential effects on plants can be connected with different hormone pathways [88,89]. Taken together, these analyses indicate that different chemical agents affect the expression of the *TDP1α* and *TDP1β* genes in distinct ways, possibly pointing to their involvement in particular processes.

The expression patterns of the two *TDP1* genes were also evaluated in response to gamma irradiation (γ-ray) treatment, known to induce the accumulation of DSB and activation of the DNA damage response (DDR) pathway. The gathered data came from 6-day-old *A. thaliana* seedlings, where the Col0 wild-type and the *sog1-1* mutant were exposed to a dose of 100 Gγ (10 Gγ/min), and the plant response was followed during a 24 h time course [49]. The *sog1-1* mutant is deficient in the function of the suppressor of gamma response 1 (SOG1) gene, considered to be the master regulator of plant DDR and a functional homolog of the mammalian p53 [90,91]. As shown in the generated heatmaps, the data mining approach, provided as a fold change relative to control data, revealed the rapid upregulation (at 20 min after treatment) of both genes in response to γ-rays in wild-type seedlings, with *TDP1α* being more expressed than *TDP1β* (Figure 5b). Moreover, in the *sog1-1* mutant, the highest expression level was observed at 20 min after the treatment, but, in this case, the *TDP1β* gene was more expressed than *TDP1α* (Figure 5c). The results indicate that the *TDP1* genes are influenced by SOG1, while the rapid activation in response to γ-rays suggests that they are immediately recruited by the DNA repair systems, possibly through both SOG1-dependent and -independent pathways.

Considering the mined data for co-expression networks and putative protein–protein interactions (see Section 3.1), the two genes’ expression data further support the involvement of these genes in DNA damage repair and the response to genotoxic stresses, in agreement with previous publications [14,25,31].

### 3.7. Experimental System to Corroborate the Impact of γ-Ray Treatment on TDP1α and TDP1β Gene Expression

**Figure 6:**
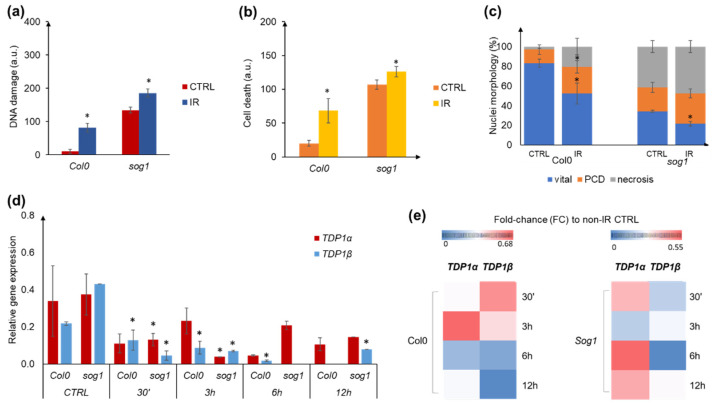
Effect of γ-ray (IR) treatments on 10-day-old *Arabidopsis* seedlings from Col0 and *sog1* mutant. (**a**) Levels of DNA damage as evidenced by comet assay and represented as arbitrary units (a.u.). (**b**) Levels of cell mortality as evidenced by diffusion assay and represented as arbitrary units (a.u.). (**c**) Percentage (%) of nuclei morphology corresponding to viable cells, programmed cell death (PCD), and necrosis events. (**d**) *TDP1α* and *TDP1β* relative gene expression at different intervals (0 h, 30 min, 3, 6, 12 h) as measured through qRT-PCR. (**e**) Gene expression data represented as fold change (FC) relative to non-irradiated control (CTRL) for each genotype. Values are expressed as mean ± SD of three replicates. Statistical significance (*p* < 0.05) is shown with an asterisk (*).

To try to verify some of the collected data, an *ad hoc* experimental system was designed similarly to the one proposed by Bourbousse et al. [49], with a few modifications. Namely, the Col0 and *sog1-1* lines were treated with the same dose of γ-rays (100 Gy), which were, however, administered to 10-day-old plantlets, subsequently sampled at 30 min, 3 h, 6 h, and 12 h timepoints for qRT-PCR analyses. However, before proceeding with the evaluation of *TDP1* gene expression, the comet and diffusion assays were carried out to evaluate the levels of DNA damage and cell death present in this system. As shown in Figure 6a, the irradiation (IR) treatment resulted in the significant accumulation of DNA damage compared with the non-treated samples (CTRL), and this accumulation was substantially higher in the *sog1* mutant compared with the Col0 lines. The same pattern was obtained when cell death events were monitored, showing enhanced levels of cell mortality being present after IR, more pronounced in the *sog1* mutant (Figure 6b). When considering the types of cellular mortality, IR induced the enhanced accumulation of programmed cell death (PCD) and necrotic nuclei in Col0, while these were more pronounced in the *sog1* mutant gene in the absence of stress (Figure 6c). These results prove once more that the *SOG1* gene deficiency is strongly correlated with defects in DNA repair activation and PCD induction [49,92,93,94]. When looking at the gene expression data, the first observation that can be made in our system is that the *TDP1β* gene seems to be more expressed in the *sog1* mutant compared to the Col0 line in the absence of IR treatment, whereas the *TDP1α* gene is similarly expressed in both genotypes (Figure 6d, CTRL samples). After 30 min following IR, both genes were downregulated compared to the non-IR CTRL, a trend that was generally maintained also at later timepoints (Figure 6d). It could be also noted that the *TDP1β* gene expression values tend to be lower than *TDP1α*. Fluctuations in gene expression could be observed also when looking at the wild-type and mutant genotypes. Hence, it can be concluded that both the *TDP1α* and *TDP1β* genes are responsive to IR treatment in a genotype-dependent manner. However, when the pattern of expression was given in terms of fold change (FC) relative to the non-IR control (Figure 6e), the trend was more similar to that gathered from the data mining approach, with the activation of genes immediately after treatment (Figure 5b,c).

The two different data representation methods (histograms versus FC) were chosen to be able to give an overall picture of the gene expression in the CTRL and IR materials, as well as to compare the patterns of expression with those obtained from the high-throughput analyses. The observed differences may be attributed not only to the different techniques used (mRNA-seq versus qRT-PCR) but also to the seedling age (5- vs. 10-day-old *A. thaliana* plantlets), together with the growth chamber conditions and slightly different timepoints for material collection.

## 4. Conclusions

**Figure 7:**
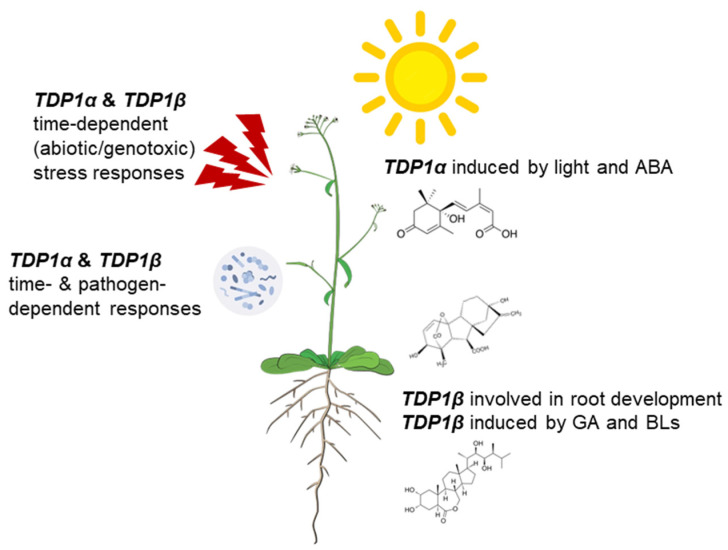
Schematic representation of the roles of the *TDP1α* and *TDP1β* genes in *A. thaliana* hypothesized based on transcriptomics data mining approaches.

In conclusion, data mining approaches prove to be a powerful tool to gather information related to poorly investigated gene functions, particularly for model plants, given the richness of transcriptomics data available. In this study, we focused on a comparative transcriptomics approach exemplified using a small gene family, composed of the *TDP1α* and *TDP1β* genes, whose diverse functions are still far from being elucidated. Even in the *A. thaliana* model plant, most studies published so far refer only to the canonical *TDP1α* gene [14,16,26], while the *TDP1β* function remains more elusive. The gene expression data gathered in this study from the Bio-Analytic Resource for Plant Biology platform allowed us to pinpoint both common and divergent functions of the two genes. A more dissected analysis related to developmental aspects and stress responses allowed us to also focus on gene-specific responses. To summarize the core of the data gathered in this study, a schematic representation of the potential roles of the two genes in *A. thaliana* is given (Figure 7).

Specifically, the presented data indicate that while *TDP1β* would appear to be more involved in root development and associated with the GA and BL phytohormones, *TDP1α* is more responsive to light and ABA. On the other hand, when considering the expression patterns under stress conditions, both genes are highly responsive to different biotic and abiotic treatments, in a time- and stress-dependent manner. Moreover, the experimental system designed to corroborate the data mining results in response to IR treatment sustains the genes’ responsiveness in a genotype-dependent manner. Further studies connecting the role of the *TDP1* genes in plant DDR are currently in progress. The hereby gathered data bring novel information related to the putative involvement of the *TDP1* genes in plant development, aside from their already known implications in the stress response, paving the way for novel research dedicated to designing additional experimental systems for further validation and in-depth characterization of these genes in plants.

## Data Availability

Not applicable.

## Supplementary Materials

The following supporting information can be downloaded at: https://www.mdpi.com/article/10.3390/genes14040884/s1. Figure S1: Protein alignment between TDP1α (AT5G15170) and TDP1β (AT5G07400) carried out with ClustalW tool; Figure S2: *Arabidopsis thaliana TDP1α* (AT5G15170) and *TDP1β* (AT5G07400) gene organization and chromosomal localization; Figure S3: *A. thaliana TDP1α* and *TDP1β* gene co-expression and co-localization networks generated using GeneMania; Figure S4: Predicted protein–protein interaction networks for *Arabidopsis thaliana* TDP1α and TDP1β sequences generated using STRING; Figure S5: Computationally predicted and experimentally documented subcellular localization of TDP1α and TDP1β proteins.
