# Supplementary material for: Integrative Transcriptomics Data Mining to Explore the Functions of TDP1α and TDP1β Genes in the Arabidopsis thaliana Model Plant
Source: Genes (Basel). 2023 Apr 9;14(4):884. doi: 10.3390/genes14040884 (PMC10137840; doi:10.3390/genes14040884)
Supplement: Supplementary file 1 [file genes-14-00884-s001.zip › genes-2319999-supplementary.pdf]

Integrative Transcriptomics Data Mining to Explore the Functions of *TDP1α* and *TDP1ß* Genes in the *Arabidopsis thaliana* Model Plant

Paola Pagano, Andrea Pagano, Stefano Paternolli, Alma Balestrazzi and Anca Macovei *

Department of Biology and Biotechnology "L. Spallanzani", University of Pavia,
27100 Pavia, Italy

* Correspondence: anca.macovei@unipv.it

```
AT5G15170      --------MAHSQVAYLIPLKADLKEDNSSPRITLSEGPNIIGRG------NVSIVDKRL
AT5G07400      MKRRQCEKVVIRIHNIGTPLISGSSGLPLELFHIQSDRPYTIGRSSSDGFCDFVIDHSSI
                       :.        ** :. .    .     *: *  ***.      :. * .. :

AT5G15170      SRKHITIIVSTSGSASLSVDGTNPVVIRSS----------------------------GD
AT5G07400      SRKHCQILFDSQSHKLYIFDGVIHLPSGSFSQVYDEFRRRLVGVEDLGNLKFRASLNGVY
               ****  *:..:..     .**.  :   *

AT5G15170      GERKKVKPSEEVSVCNDDLIELIPG-----------------------------------
AT5G07400      VNRVRVRKSKVQEVSIDDEVLFFCGKEGLCCKDGRVGFVVQEIVFEGRDASIVSVSSGHS
                :* :*: *:  .*. ** : :: *

AT5G15170      ------------------------------------------------------------
AT5G07400      RGTFSSGKRSKRVFAPMENEINSPVSGFYPPKAVGVVERVNSLVSYCRHILKSDDPLSCL


AT5G15170      ------------------------------------------------------------
AT5G07400      RLSIISHSGKECLSCCTSKMFRSKVGIVADDRGVKSAEINHDMGHGLSGLRLSIERPNSN


AT5G15170      -----------------HHFFKLVLLNGRAAKKARKAEDDVEAIRRFCPPNEK-------
AT5G07400      LHVDRRLGVSDLISEIENEFAACTFISDKTRTMLPFDGEKVNTPDITCINKEKSYQSSLQ
                                :.*   .::..:: .      :.*::    *  :**

AT5G15170      LPSTFRLLSVDALPDWANTSCVSINDVIEG-----DVVAAILSNYMVDIDWLMSACPKLA
AT5G07400      APGKNFYLNRLQYIEQSSTGCQRVVSLPELLHPVESIQQIFLATFTSDILWFLTCCDTPR
                *..   *.     : :.*.*  : .: *      .:   :*:.:  ** *:::.* .

AT5G15170      NIPQVMVIHGEG-------DGRQEYIQRKKPANWILHKPR-LPISFG---------THHS
AT5G07400      HLPVTIACHNAERCWSSNPDARTAVPLPNYPNVTMVYPPFPEEIAFGKDRTNRGIACHHP
               ::* .:. *.         *.*      : *   ::: *    *:**          **.

AT5G15170      KAIFLVYPRGVRVVVHTANLIHVDWNNKSQGLWMQDFPWKDDDKDPPKGCGFEGDLIDYL
AT5G07400      KLFILQRKDSIRVIITSANLVARQWNDVTNTVWWQDFPRRAD----PDLLSLFGHCQRET
               * ::*    .:**:: :***:  :**: :: :* **** : *    *.  .: *.

AT5G15170      NVLKWPEFTANLPGRGNVKIN--------AAFFKKFDYSDATVRLIASVPGYHTG-----
AT5G07400      NHGLKPDFCAQLAGFAASLLTDVPSQAHWILEFTKYNFEHSAGHLVASVPGIHSYKPSYL
               *    *:* *:*.* .   :.           *.*::..::  :*:***** *:

AT5G15170      ------------------------------------------------------------
AT5G07400      TESGCSNTIFSEEFLGSVEASVVGLSYLFRSANDSTGAQLKRLASYIRRTRENSLGMLEL


AT5G15170      ----------------------------------------------------FNLNKWGH
AT5G07400      VLRRNTNVPADPNAVRVLVPNPDDDSRDDFVQLGFLPRSIAKWVSPLWDIGFFKFVGYVY
                                                                   *::  : :

AT5G15170      MKLRTILQECIFDREFRRSPLIYQFSSLGSLDEKWLAEFGNSLSSGITEDKTPLGPG---
AT5G07400      RDEVLGAASCRSNEKVQLVLHVLQGVSISDMSKLIQPYHVVALCSLIASLQRCTGIWRLQ
                .      .*  :.:.:    : *  *:..:.:   . .  :*.* *:. :   *

AT5G15170      --DSLIIWPTVEDVRCSLEGYAAGNAIPS-------------------------------
AT5G07400      EVLGRYKWPESQESDFVYSASSIGGSATTGFQADFSSAAGKKALQHFDSQESDPEWGCWS
                  .   **  ::     .. : *.: .:

AT5G15170      -------PLKNVEKP--------------------FLKKYWARWKAD-------------
AT5G07400      NREEREAPSIKIIFPTIERVKNGHHGVLSSRRLLCFSEKTWQKWRHSNVLHDAVPNPQDR
                      *  ::  *                    * :* * :*: .

AT5G15170      --HSARGRAMPHIKTFTRYN-DQKIAWFLLTSSNLSKAAWGALQKNNSQ-----------
AT5G07400      VGHPMHIKVARRLFTSTRGSRSSSFGWVYSGSHNFSAAAWGQTISRSSRNNQDQSNNAIR
                 *. : :.  :: * ** . ...:.*.   * *:* ****   ...*:

AT5G15170      ----LMIRSYELGVLFLPSP----------IKTQGCVFSCTESNPSVMKAKQETKDEVEK
AT5G07400      AVKKLRVCNYELGIVFVFPPPHEETDSCEGSKIDDIVLPFVVPAPKYGWSDKPATGLAMR
                   * : .****::*: .*           * :. *:. . . *.   :.: :.. . :

AT5G15170      RSKLVTMTWQGDRDLPEIISLPVPYQLPPKPYSPEDVPWSWDRGYSKKDVYGQVWPR---
AT5G07400      EAFAEFREGSTSFCGESEVEEEVEEEEEEEADAEGRGEFVAEEEKQEEEAYAEALWSQVE
               .:       . .    . :.   *  :   :. :     :  :.  .:::.*.:.

AT5G15170      --------
AT5G07400      SSSSSLSS
```

Supplementary Figure S1. Protein alignment between TDP1α (AT5G15170) and TDP1β (AT5G07400) carried out with ClustalW tool. Main protein domains are evidenced as follows, the FHA (Fork-associated domain) in blue, the two HKD catalytic domains, structured as

aHxK(x)4D(x)6GSxN sequence, in green, and the HIRAN domain, present only in the TDP1β sequence, in dark red. The sequence similarity calculations indicate 123 aa conserved indicating relatively 11% similarity between the two protein sequences.

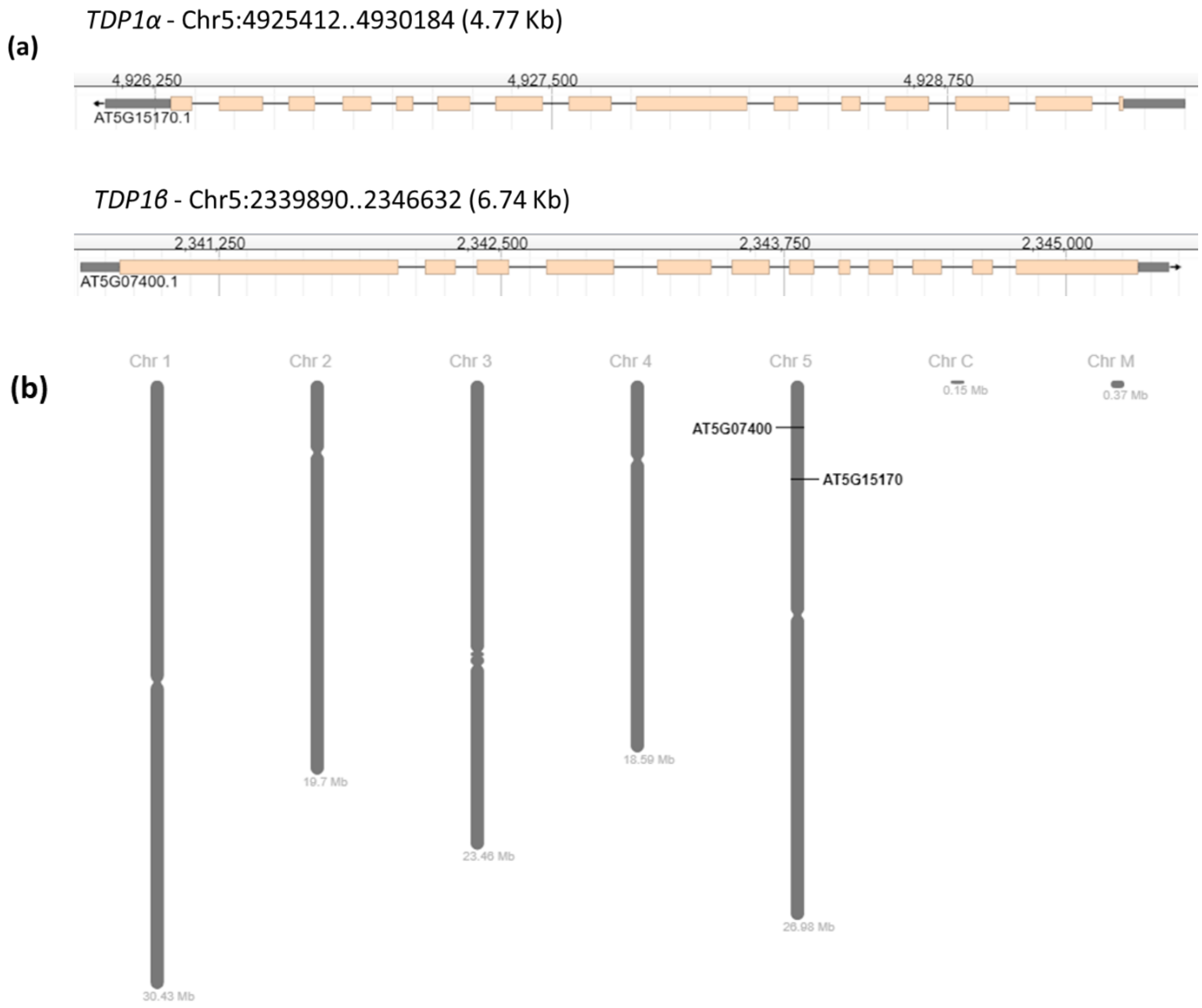

Supplementary Figure S2. *Arabidopsis thaliana TDP1α* (AT5G15170) and *TDP1β* (AT5G07400) gene organization **(a)** and chromosomal localization **(b)**.

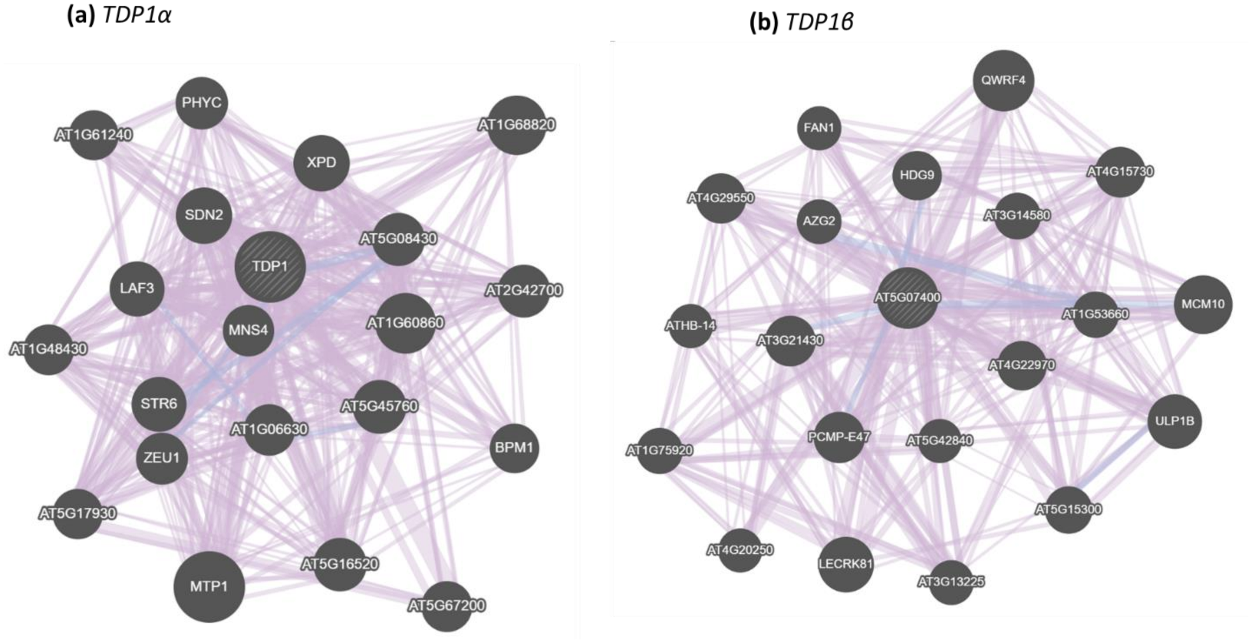

Supplementary Figure S3. *Arabidopsis thaliana TDP1α* and *TDP1β* gene co-expression and colocalization networks generated using GeneMania (https://genemania.org/). Co-expression with other genes are given with violet lines while co-localization data is shown with blue lines. **(a)** *TDP1α* co-expressed genes brief description: *AT1G61240*, lysine ketoglutarate reductase trans-splicing-like protein (DUF707), involved in temperature-dependent root growth; *AT1G68820*, PPRT1, putative C3HC4 zinc-finger ubiquitin E3 ligase, negative regulator in ABA and drought stress response; *AT1G48430*, dihydroxyacetone kinase, required for glycerolipid metabolism and for the activation of systemic acquired resistance; *AT5G08430*, SWIB/MDM2 and Plus-3 and GYF domain-containing protein, involved in plant-pathogen interactions; *AT1G60860*, AGD2, VAN3-LIKE PROTEIN2 involved in plant defense and senescence; *AT1G42700*, hypothetical protein, unknown function; *AT1G06630*, F-box/RNI-like superfamily protein involved in cell division and during the transition from skotomorphogenesis to photomorphogenesis; *AT5G45760*, transducin/WD40 repeat-like superfamily protein, involved in chromatin assembly; *AT5G17930*, MIF4G domain-containing protein/MA3 domain-containing protein; *AT5G16520*, transmembrane protein; *AT5G67200*, leucine-rich repeat protein kinase family protein; *PHYC*, PHYTOCHROME C family of photoreceptors that modulate plant growth and development; *XPD/UVH6*, general transcription and DNA repair factor IIH helicase subunit XPD *Xeroderma pigmentosum*, role in response to light and DNA damage/repair; *SDN2*, small RNA degrading nuclease 2; *LAF3*, protein LONG AFTER FAR-RED 3, required for phyA-controlled responses to continuous far-red light (FRc) conditions, including the inhibition of hypocotyl elongation; *MNS4*, MANNOSIDASE 4, glycosyl hydrolase family 47 protein involved in the degradation of misfolded variants of the heavily glycosylated brassinosteroid receptor, BRASSINOSTEROID INSENSITIVE1; *STR6*, rhodanese-like domain-containing protein 6 involved in signaling processes as sulfur carriers, required for anchoring ferredoxin-NADP reductase to the thylakoid membranes and sustaining efficient linear electron flow (LEF); *ZEU1*, thymidine kinase 1, involved in tolerance to genotoxic stress; *BPM1*, encodes a member

of the MATH-BTB domain proteins that directly interact with and target for proteasomal degradation the class I homeobox-leucine zipper (HD-ZIP) transcription factor ATHB6; *MTP1*, metal tolerance protein 1,mediates zinc accumulation in roots and confers resistance to zinc. **(b)** *TDP1β* co-expressed genes brief description: *AT4G29550*, hypothetical protein (DUF626); *AT3G14580*, Pentatricopeptide repeat (PPR) superfamily protein, involved in biotic stress; *AT4G15730*, CW-type Zinc Finger; *AT3G21430*, EARLY 3, DNA binding protein; *AT1G53660*, nucleotide/sugar transporter family protein; *AT4G75920*, secE/sec61-gamma protein transport protein; *AT1G75920*, EXL5, GDSL-motif esterase/acyltransferase/lipase; *AT5G42840*, Cysteine/Histidine-rich C1 domain family protein, involved in plant defense response; *AT4G20250*, hypothetical protein; *AT3G13225*, WW domain-containing protein involved in viral defense; *AT5G15300*, PPR superfamily protein; *FAN1*, nuclease involved in DNA crosslink repair; *HDG9*, HOMEODOMAIN GLABROUS 9, encodes a homeobox-leucine zipper family protein belonging to the HD-ZIP IV family; *HDG2*, homeodomain GLABROUS 2; *ATHB-14*, homeobox-leucine zipper protein ATHB-14; *QWRF4*, QWRF motif-containing protein 4; *AZG2*, adenine/guanine permease involved in the transport of purines; *MCM10*, MINICHROMOSOME MAINTENANCE 10, involved in the initiation of DNA replication; *PCMP-E47*,PPR superfamily protein; *ULP1B*, UB-like protease 1B involved in biotic stress; *LECRK81*, L-TYPE LECTIN RECEPTOR KINASE II.1, involved in plant insect defense.

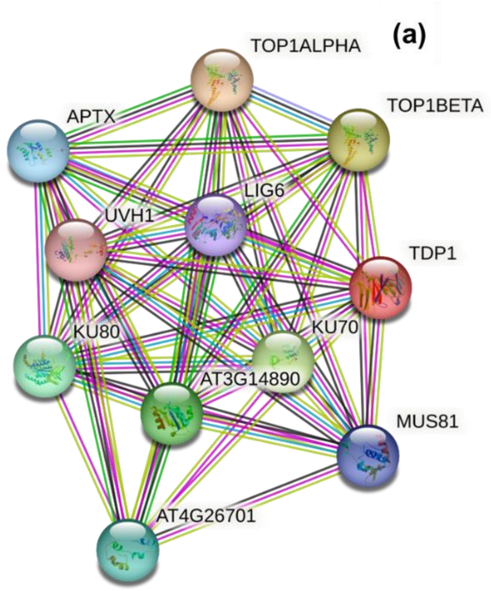

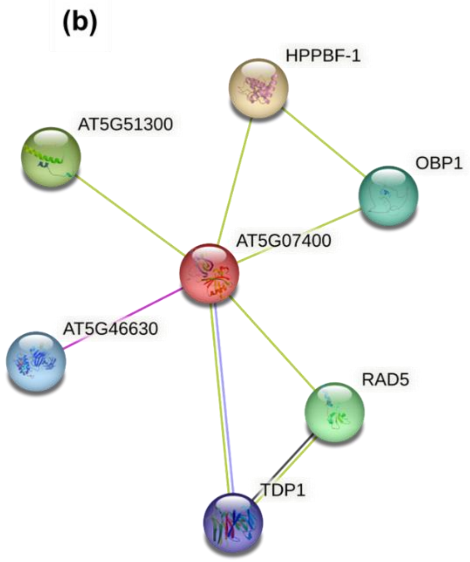

Supplementary Figure S4. Predicted protein-protein interaction networks for *Arabidopsis thaliana* TDP1α and TDP1β sequences generated using STRING (https://string-db.org/). Line color legend: turquoise, data from curated databases; viola, experimentally determined; green, gene neighborhood; red, gene fusion; dark blue, gene co-occurrence; brown, text-mining; black, co-expression; light blue, protein homology. **(a)** TDP1α predicted interactors: AT3G14890, bifunctional polynucleotide phosphatase/kinase; Polynucleotide 3'-phosphatase ZDP; Nick-sensing 3'-phosphoesterase involved in a base excision repair pathway required for active DNA

demethylation; AT4G26701, DNA binding, DNA topoisomerase type I, involved in DNA topological change, DNA unwinding involved in replication; TOP1ALPHA, DNA topoisomerase I alpha, releases the supercoiling and torsional tension of DNA introduced during the DNA replication and transcription by transiently cleaving and rejoining one strand of the DNA duplex; TOP1BETA, DNA topoisomerase I beta; APTX, APRATAXIN-LIKE, transcription factor bHLH140 with adenylyl sulfate sulfohydrolase activity; UVH1, restriction endonuclease, type II-like superfamily protein, involved in NER, the repair of UV light, and oxidative damage; LIG6, DNA ligase 6, seals nicks in dsDNA during DNA replication, DNA recombination and DNA repair, required to maintain seed viability and during seed germination; KU70, ATP-dependent DNA helicase 2 subunit KU70, ssDNA-dependent ATP-dependent helicase, involved in NHEJ required for DSB repair; KU80, ATP-dependent DNA helicase 2 subunit KU80, forms a heterodimer with KU70, the KU70/80 complex, required for proper maintenance of the telomeric C strand; MUS81, restriction endonuclease, type II-like superfamily protein involved in DPC repair in independent pathways to WSS1A and TDP1. **(b)** TDP1β predicted interactors: AT5G51300, splicing factor-like protein, required for the splicing of pre-mRNA during plant development and in response to ABA; AT5G46630,clathrin adaptor complexes AP-2medium subunit family protein, functions in protein transport via transport vesicles in different membrane traffic pathways; HPPBF-1, telomere repeat-binding protein 4, it binds specifically to the plant telomeric dsDNA sequences 5'-TTTAGGG-3'; OBP1, Dof zinc finger protein DOF3.4, transcription factor that binds specifically to a 5'- AA[AG]G-3' consensus core sequence and enhances the DNA binding of OBF transcription factors to OCS elements; RAD5, DNA repair protein RAD5A, functions in error-free post-replication DNA repair or DNA-damage tolerance (DTT) pathway, required for HR-induced by DSBs; TDP1, tyrosyl-DNA phosphodiesterase-like protein, DNA repair enzyme that catalyzes the hydrolysis of dead- end complexes between DNA and the topoisomerase I active site tyrosine residue.

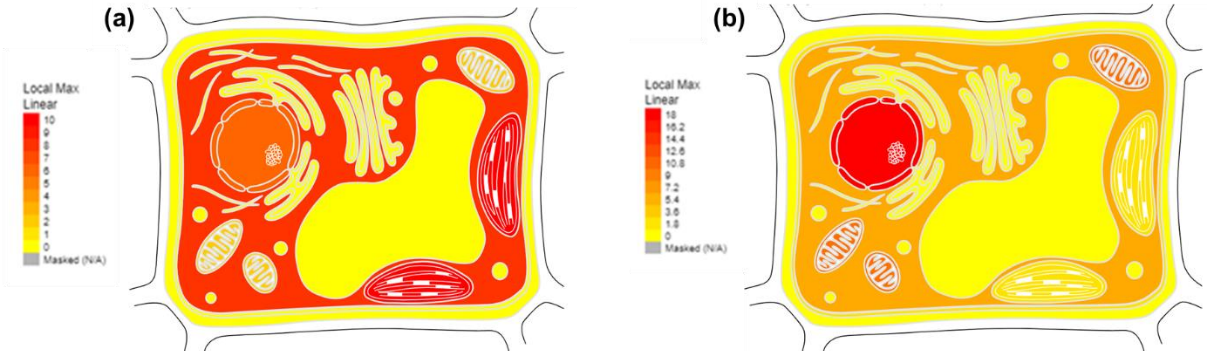

Supplementary Figure S5. Computationally predicted and experimentally documented subcellular localization of the *Arabidopsis thaliana* TDP1α **(a)** and TDP1β **(b)** proteins as obtained from ePlant (http://bar.utoronto.ca/eplant). The higher the confidence score for a given subcellular compartment, the more intense the red color in the Cell eFP Browser output.
